# Supplementary figures and images for: Splicing factor SRSF1 is essential for homing of precursor spermatogonial stem cells in mice
Source: eLife. 2024 Jan 25;12:RP89316. doi: 10.7554/eLife.89316 (PMC10945694; doi:10.7554/eLife.89316)

Figure 1C

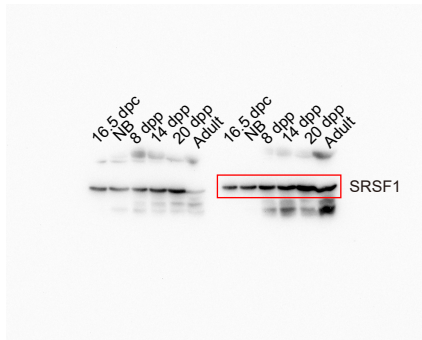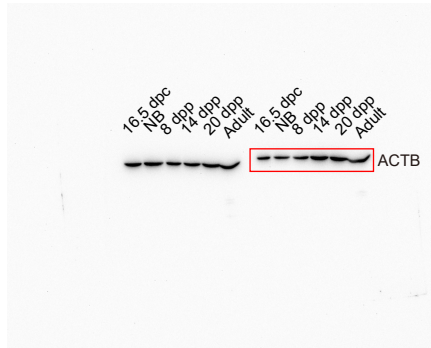

Supplement: Figure 1—source data 1. — ACTB served as a loading control. [file elife-89316-fig1-data1.pdf]

Figure 3B

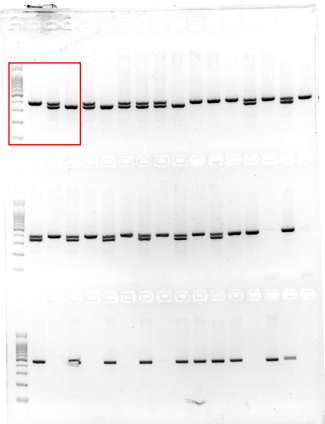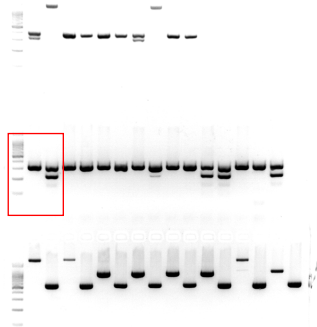

Supplement: Figure 3—source data 1. [file elife-89316-fig3-data1.pdf]

Figure 7B

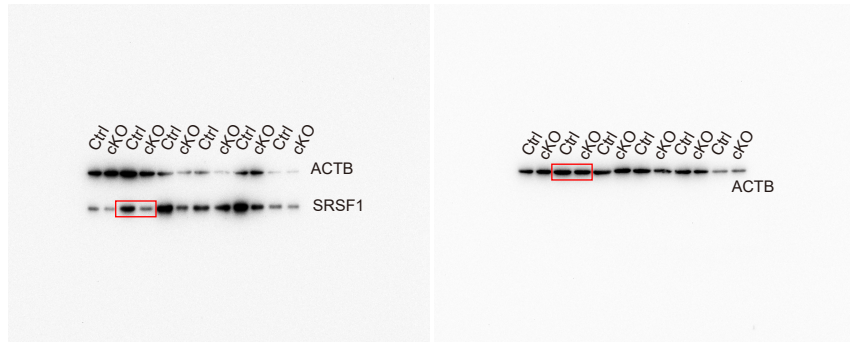

Supplement: Figure 7—source data 1. — ACTB served as a loading control. [file elife-89316-fig7-data1.pdf]

Figure 8E

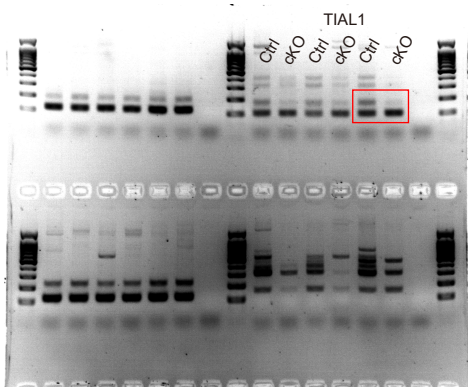

Supplement: Figure 8—source data 1. [file elife-89316-fig8-data1.pdf]

Figure 8J

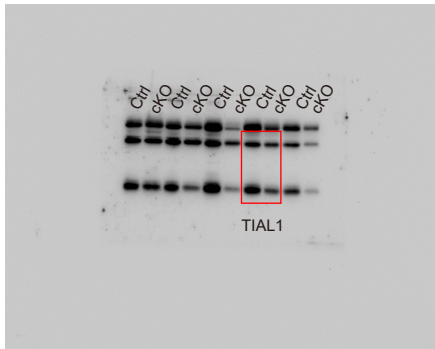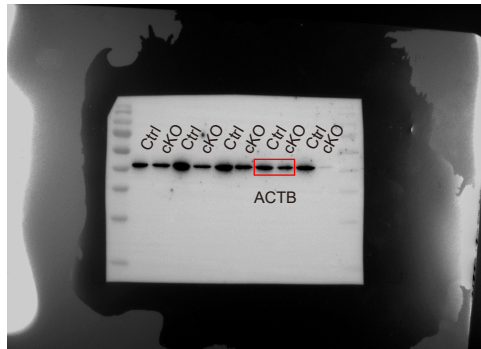

Supplement: Figure 8—source data 2. — ACTB served as a loading control. [file elife-89316-fig8-data2.pdf]

Figure 9B

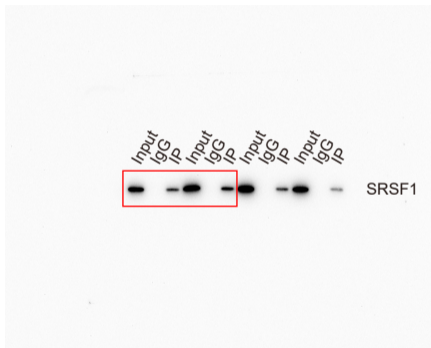

Supplement: Figure 9—source data 1. [file elife-89316-fig9-data1.pdf]

Figure 9I

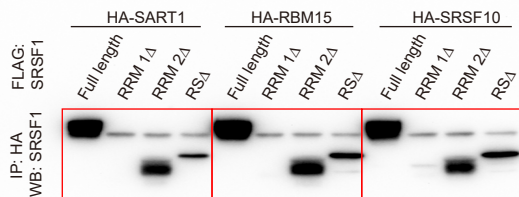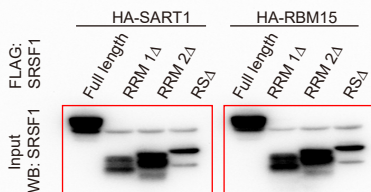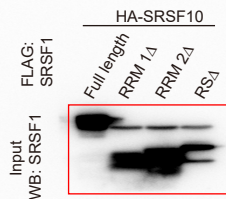

Supplement: Figure 9—source data 3. [file elife-89316-fig9-data3.pdf]
