# Supplementary material for: Splicing factor SRSF1 is essential for homing of precursor spermatogonial stem cells in mice
Source: eLife. 2024 Jan 25;12:RP89316. doi: 10.7554/eLife.89316 (PMC10945694; doi:10.7554/eLife.89316)
Supplement: Figure 9—source data 2. [file elife-89316-fig9-data2.pdf]

Figure 9G

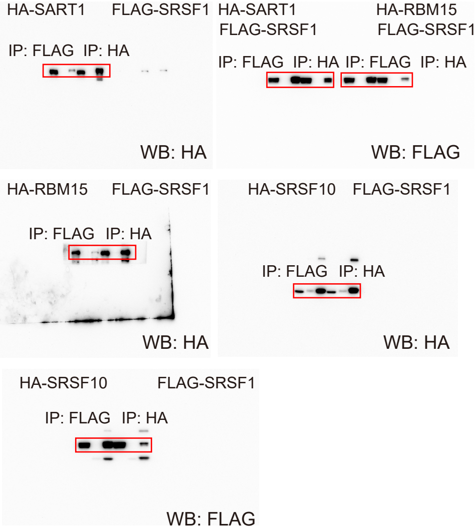

HA-SART1
FLAG-SRSF1
IP: FLAG
IP: HA
WB: HA
HA-SART1
FLAG-SRSF1
HA-RBM15
FLAG-SRSF1
IP: FLAG
IP: HA
IP: FLAG
IP: HA
WB: FLAG
HA-RBM15
FLAG-SRSF1
IP: FLAG
IP: HA
WB: HA
HA-SRSF10
FLAG-SRSF1
IP: FLAG
IP: HA
WB: HA
HA-SRSF10
FLAG-SRSF1
IP: FLAG
IP: HA
WB: FLAG
